# Exploring Aesthetic Perception in Impaired Aging: A Multimodal Brain—Computer Interface Study

**DOI:** 10.3390/s24072329

**Published:** 2024-04-06

**Authors:** Livio Clemente, Marianna La Rocca, Giulia Paparella, Marianna Delussi, Giusy Tancredi, Katia Ricci, Giuseppe Procida, Alessandro Introna, Antonio Brunetti, Paolo Taurisano, Vitoantonio Bevilacqua, Marina de Tommaso

**Affiliations:** 1 Translational Biomedicine and Neuroscience (DiBraiN) Department, University of Bari, 70124 Bari, Italy; livio.clemente@uniba.it (L.C.); giulia.paparella@uniba.it (G.P.); marianna.delussi@uniba.it (M.D.); tancredi.giusy.96@gmail.com (G.T.); katiari86@gmail.com (K.R.); beppepine@gmail.com (G.P.); ale.introna@gmail.com (A.I.); paolo.taurisano@uniba.it (P.T.); 2 Interateneo Department of Fisica ‘M. Merlin’, University of Bari, 70125 Bari, Italy; marianna.larocca@uniba.it; 3 Laboratory of Neuroimaging, USC Stevens Neuroimaging and Informatics Institute, Keck School of Medicine of USC, University of Southern California, Los Angeles, CA 90033, USA; 4 Electrical and Information Engineering Department, Polytechnic of Bari, 70125 Bari, Italy; antonio.brunetti@poliba.it (A.B.); vitoantonio.bevilacqua@poliba.it (V.B.)

**Keywords:** BCI, aesthetic, fNIRS, EEG, impaired aging

## Abstract

In the field of neuroscience, brain–computer interfaces (BCIs) are used to connect the human brain with external devices, providing insights into the neural mechanisms underlying cognitive processes, including aesthetic perception. Non-invasive BCIs, such as EEG and fNIRS, are critical for studying central nervous system activity and understanding how individuals with cognitive deficits process and respond to aesthetic stimuli. This study assessed twenty participants who were divided into control and impaired aging (AI) groups based on MMSE scores. EEG and fNIRS were used to measure their neurophysiological responses to aesthetic stimuli that varied in pleasantness and dynamism. Significant differences were identified between the groups in P300 amplitude and late positive potential (LPP), with controls showing greater reactivity. AI subjects showed an increase in oxyhemoglobin in response to pleasurable stimuli, suggesting hemodynamic compensation. This study highlights the effectiveness of multimodal BCIs in identifying the neural basis of aesthetic appreciation and impaired aging. Despite its limitations, such as sample size and the subjective nature of aesthetic appreciation, this research lays the groundwork for cognitive rehabilitation tailored to aesthetic perception, improving the comprehension of cognitive disorders through integrated BCI methodologies.

## 1. Introduction

The field of neuroscience is constantly evolving in both research and healthcare, and a major role in this process is assumed by the brain–computer interface (BCI) [1,2]. BCIs are advanced systems that enable direct communication between the human brain and external devices [3], and their efficacy has been demonstrated in multiple settings, from detection to intervention. The main goal of BCI research is to collect the most accurate real-time data on brain activity in the simplest and least invasive way, with the shortest calibration and set-up time [4]. Studies have shown significant improvements in motor function through the use of BCIs, exceeding the performance of traditional control therapies and highlighting its potential in post-stroke motor rehabilitation [5,6]. Additionally, it has been noted that adding local network features to BCIs based on steady-state visual evoked potentials (SSVEPs) can greatly increase the information transfer rate and classification accuracy, providing new opportunities for BCI optimization of performance across a broad spectrum of application domains [7]. The integration of BCIs with other technologies, such as neural signal analysis and data processing, is improving the accuracy and effectiveness of these systems. For example, the use of advanced machine learning algorithms [8] and brain connectivity [9] has refined the decoding of neural signals, improving the interface between the brain and external devices.

To classify and understand the wide range of BCI technologies available, an important distinction is made between invasive and non-invasive systems, each with distinct characteristics and application areas [10]. Invasive BCI systems, which require surgical implantation of electrodes in the brain, including intracortical electrodes [11] and electrocorticography (ECoG) [12], offer high-quality signals with high spatial and temporal resolution, making them particularly useful in research settings and for applications that require very precise control [10]; however, these systems carry greater risks and complications related to the surgical procedure [13].

Non-invasive BCIs capture neural signals directly from the surface of the skull using devices such as electroencephalography (EEG), functional near-infrared spectroscopy (fNIRS), functional magnetic resonance imaging (fMRI), magnetoencephalography (MEG), and positron emission tomography (PET), without the need for invasive surgery [14]. These methods provide unprecedented access to brain activity and enable advanced communication between the brain and external devices in a safe and non-invasive manner. However, MEG, PET, and fMRI are still technically demanding and expensive [15].

In the context of neurosciences, these non-invasive techniques are fundamental for the study of central nervous system activity, and among them, EEG emerges as the most widely used method for measuring electrical brain activity [16]. EEG is particularly prized for its portability, non-invasiveness, and, most importantly, its ability to monitor brain activity with high temporal resolution, making it one of the most effective techniques in this field [17]. In EEG signals, it is possible to identify so-called evoked potentials, which are specific neurophysiological responses measured in reaction to standardized and known sensory stimuli. These evoked potentials provide a functional assessment of sensory systems, including auditory, visual, and somatosensory [18]. The various categories of evoked potentials, which include event-related evoked potentials (ERPs), visual evoked potentials (VEPs) [19], acoustic evoked potentials (AEPs), motor evoked potentials (MRPs), and steady-state visual evoked responses (SSVEPs) [20], are fundamental tools for reflecting brain activity in response to specific sensory stimuli and find application in the monitoring and diagnosis of various neurological disorders in clinical and domestic settings.

Similarly, fNIRS is praised for its ability to monitor cognitive activity by measuring task-related hemodynamic responses in the prefrontal cortex, providing a unique window into brain function during mental tasks [21,22]. Recent studies have shown that fNIRS can be effectively used to record signals from cognitive, visual, and auditory functions [20,23]. This significantly expands the field of application of BCIs, allowing not only the improvement of motor functions as demonstrated in post-stroke rehabilitation [5,6] but also the exploration of new frontiers in the monitoring and intervention of complex cognitive functions. The ability to detect responses related to problem-solving tasks and activities requiring concentration and memory underscores the adaptability of fNIRS to a variety of BCI applications, making the signals not only easily monitored by users, but also closely related to the underlying cognitive activity [24].

Neuroscience research has investigated the impact of visual stimuli, both static and dynamic, on the motor areas of the brain during aesthetic perception [25]. Studies have shown that viewing dynamic works of art, such as those by van Gogh, not only enriches the perceptual experience through complex details but also activates movement-related brain areas, such as the M+ area [26]. These findings suggest a link between aesthetic perception and motor processing [27]. Research suggests that observing static human actions involved in motion can increase cortical activation. The brain areas involved in this process include V5/MT, EBA, and motor cortices [28]. Therefore, it is important to consider the motor area when discussing aesthetic appreciation.

The combination of EEG and fNIRS offers new directions for a comprehensive analysis of brain function by integrating electrical and hemodynamic data [29]. This integrated approach, EEG-fNIRS, has demonstrated its value in various areas of neuroscience, providing a holistic perspective on brain functioning [30].

With this in mind, the purpose of this study is to explore aesthetic perception in individuals with mild impairment using a multimodal approach that combines EEG and fNIRS in the context of non-invasive BCI technologies. The study investigates how changes in brain activity, as measured through these two methodologies, may reflect differences in response to aesthetic stimuli. Our study aims to provide new insights into the underlying neural dynamics and offer suggestions for the development of more effective diagnostic and therapeutic tools by analyzing responses evoked by aesthetic stimuli. We will contribute to exploring the potential of BCI technologies in clinical and research contexts.

## 2. Materials and Methods

### 2.1. Study Participants

**Figure 1:**
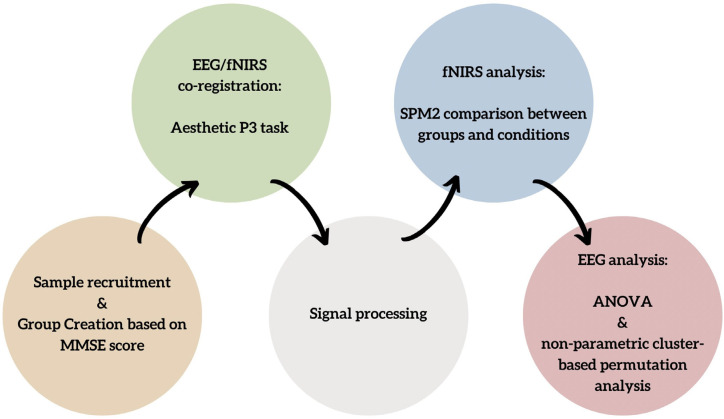
Flow chart of the study design illustrating the stages of recruitment, recording, data processing, and group comparison.

Twenty participants were recruited from the Psychology and Clinical Neuropsychology Clinic and the U.O.C. University Neurophysiopathology Unit of Bari General Hospital. All participants had a Mini-Mental State Examination (MMSE) score greater than 18 to exclude moderate to severe cognitive impairment [31]. The sample was then divided according to their MMSE score (Figure 1), creating two groups of ten subjects each: a clinical group with MMSE scores from 18 to 23, indicating impaired aging (IA), and a control (CT) group with MMSE scores from 24 to 30 [31]. Healthy and impaired aging groups did not differ in terms of age (*t*(18) = −0.67, *p* = 0.510).

The study was conducted from May 2022 to February 2023. All participants were right-handed and older than 65 years of age. The experimental procedures were approved by the ethics committee of the General Polyclinic of Bari and were performed in accordance with the Declaration of Helsinki. Participants were informed of the purpose of the study before the experiment and provided written informed consent. None of the subjects in this study had any prior experience with the recording devices or the experimental task, and they were all able to independently follow the study’s instructions.

### 2.2. Recording Techniques

The cerebral hemodynamic and bioelectrical activity was recorded using an EEG-fNIRS co-recording cap with an additional black cap placed over it to block out any potential ambient light interference (Figure 1).

The EEG cap consisted of 61 encephalic EEG channels positioned according to the enlarged international standard 10–20 system, as shown in Figure 1 (Fp1, FPZ, Fp2, F7, F3, Fz, F4, F8, T3, C3, Cz, C4, T4, T5, P3, Pz, P4, T6, O1, Oz, O2, AF7, AF3, AFz, AF4, AF8, F5, F1, F2, F6, FT7, FC5, FC3, FC1, FCz, FC2, FC4, FC6, FT8, C5, C1, C2, C6, TP7, CP5, CP3, CP1, CPz, CP2, CP4, CP6, TP8, P5, P1, P2, P6, PO7, PO3, POz, PO4, and PO8). A biauricular reference electrode was used. In addition, to remove any ocular blink artifacts, two electrooculogram detection electrodes were applied at the level of the outer canthus of the right and left eye, while the ground electrode was placed on the right forearm. The impedance was kept below 5 KW. During the EEG recording, we used a digital filter in the 0.1–70 Hz range and a 50 Hz notch filter to allow signal inspection.

**Figure 2:**
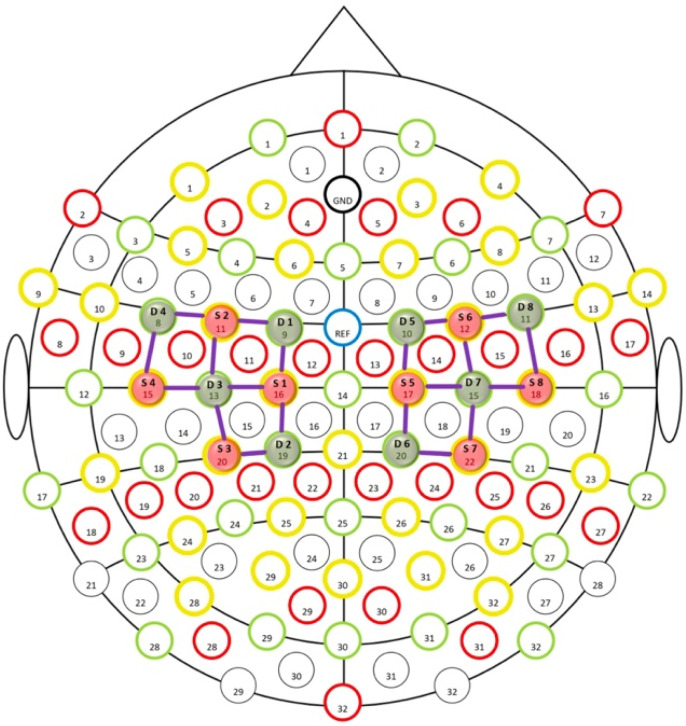
Combined EEG/fNIRS system with 10/20 64 electrodes and 20 NIR channels resulting from 16 optodes (8 sensors in red and 8 detectors in green).

fNIRS sensors were placed on the EEG headset, allowing the simultaneous acquisition of both parameters (Figure 2). The continuous wave fNIRS system (NIRSport 8 8, Nirx Medical Technologies LLC, Berlin, Germany) was used to conduct the current investigation. The fNIRS device consists of a multi-channel system able to measure hemodynamic activity fluctuations. For data acquisition, NIRStar 14.2 software was adopted (Version 14, Revision 2, Release Build, 15 April 2016 NIRx Medizintechnik GmbH, Berlin, Germany). The easy-to-use device involved light-emitting diode (LED) sources and photosensitive detectors (sensitivity: >1 pW, dynamic range: >50 dB). More exactly, the data were recorded by eight light sources, from which lights were emitted from each source at two different wavelengths (760 nm and 850 nm), and eight detectors. The transmitter–receiver distance was 30 mm, as recommended in the scientific literature [32]. Sources, detectors, and the layout of the 20 fNIRS channels were located as illustrated in Figure 1. For these specific registrations, the probes were placed on the cap over the primary motor cortex (M1) and supplementary motor cortex. The sampling rate was 7.81 Hz. The optical density was converted to variations in oxyhemoglobin (HbO_2_) and deoxyhemoglobin (HbR), using the modified Beer–Lambert law (mBLL) [33]. Before each fNIRS recording, a calibration procedure was employed to determine the signal amplification required for every source–detector pattern. 

#### 2.2.1. Paradigm

**Figure 3:**
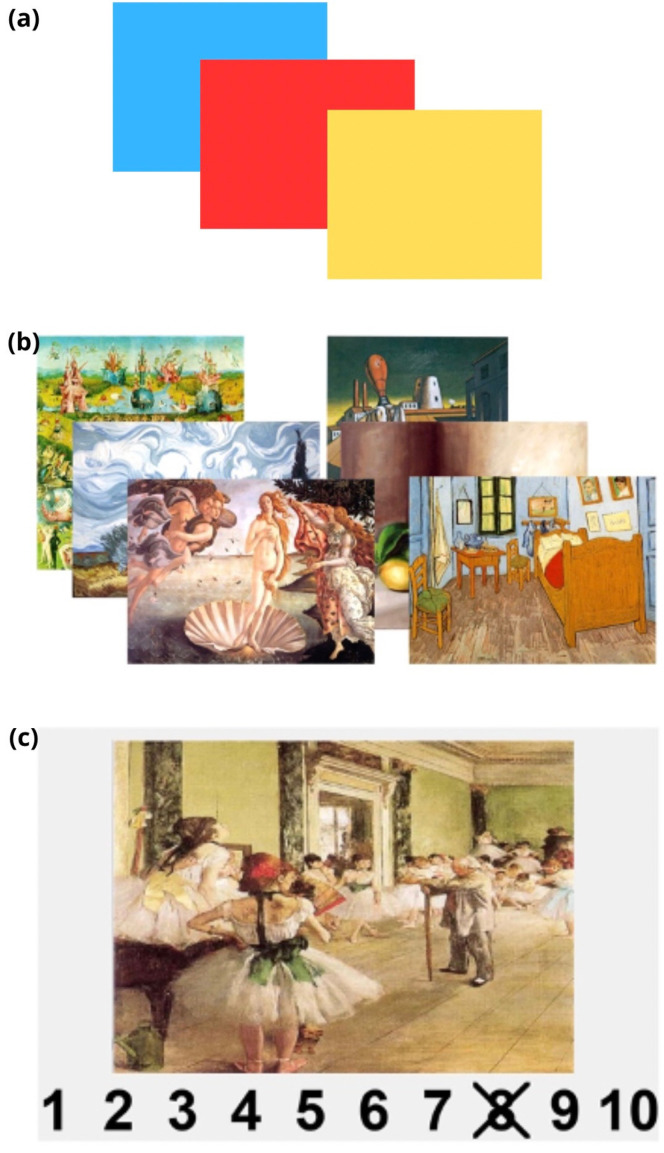
Experimental paradigm, showing (**a**) common stimuli; (**b**) target dynamic stimuli on the left side and target static stimuli on the right; (**c**) Likert scale for evaluation of aesthetic appreciation.

Participants were examined in a well-ventilated room and positioned in front of a screen in an ergonomic position. The tasks included an initial two-minute resting state baseline, in which the subject was asked to fix a cross in the center of the black screen. The methodology adopted consisted of presenting an odd-ball paradigm to the subjects in which frequent and rare stimuli were alternated. A total of 165 visual stimuli were selected, divided into 115 single-color frequent images and 50 target images, consisting of 25 static and 25 dynamic aesthetic stimuli (Figure 3). Each visual stimulus had a duration of 5 s, and the inter-stimulus interval was 9 s. The frequency of occurrence of the standard stimulus was 70%, while for the target stimulus, it was 30%. The motor task at the appearance of the target stimulus (static or dynamic images) consisted of pressing, as quickly as possible, the space bar of a PC keyboard provided to the participant. This keyboard was triggered on the EEG and NIRS trace, and each time it was pressed, an indicator marker appeared on the trace. At the end of the task, participants were asked to rate each image on a Likert scale from 1 to 10 based on their aesthetic appreciation.

#### 2.2.2. fNIRS Data Processing

Data preprocessing was performed using NirsLAB v.2019.04 software. Before data preprocessing, the images were separated into different files according to the level of attractiveness of the images for each subject, dividing into separate files the images that each subject had considered attractive (scores of 7 or higher), neutral (scores of 5 and 6), and unattractive (scores of 4 or lower). Markers were then set to indicate the onset of each condition (common stimulus, target static image, target dynamic image, and resting state period), with a stimulus duration of 14–14–14–120 s, respectively. For fNIRS data preprocessing, discontinuities were removed (STD threshold = 5) and channels were interpolated (maximum frames = 4). Subsequently, the signals recorded in each channel were inspected by removing channels containing sufficient noise (gain setting > 8; coefficient of variation > 7.5), and a bandpass filter of 0.01–0.2 Hz was applied. The hemodynamic states were then calculated for each channel, and the Beer–Lambert law was used to convert the processed signals into variations of oxygenated and deoxygenated hemoglobin concentrations (baseline in frames = 39–195, corresponding in 5–25 s of baseline).

During our fNIRS analysis, we specifically observed the changes in oxyhemoglobin (HbO) triggered by the P300 test. Our analysis focused on stimulus-related changes within a 14-s time window after stimulus onset. This enabled us to capture the immediate hemodynamic dynamics evoked by the different levels of image attractiveness assessed by the subjects.

### 2.3. EEG Data Processing

The EEG data analysis was performed using an automated workflow based on EEGLAB (version 2022) in MATLAB. We applied an FIR filter to limit the frequency range between 1 and 30 Hz. Next, we implemented the Artifact Subspace Reconstruction (ASR) method to correct the continuous data by discarding unsuitable channels and data segments. We then interpolated the bad channels and recalibrated the entire dataset to the mean. For each 0.5-s time window, we set a maximum deviation in the standard deviation of 20. In addition, we eliminated channels that were inactive for more than 5 s, those with a high-frequency noise standard deviation of less than 4, and those with a correlation greater than 0.8 with adjacent channels. Next, an independent component analysis (ICA) was performed, and artifactual components were automatically excluded through the use of a machine learning algorithm called the Multiple Artifact Rejection Algorithm (MARA). Components with a greater than 50% probability of being artifacts were removed. Finally, the data were divided into epochs in the time interval from −0.3 s to 1 s, and the baseline was corrected.

### 2.4. Statistical Methods

#### 2.4.1. fNIRS

To conduct an in-depth investigation of the brain areas involved, we performed a topographical analysis using the Generalized Linear Model (GLM) based on Statistical Parametric Mapping for fNIRS (NIRS-SPM), implemented via SPM 8 software in NirsLAB. This approach enabled us to accurately identify the brain regions that were active during task execution for each case. The study employed the Hemodynamic Response Function (HRF) to represent the hemodynamic response to the experimental tasks in the Statistical Parametric Mapping analysis (SPM1, intra-subject). The degree of activation of each channel relative to the baseline was calculated via the beta value.

The study analyzed the differences between subjects using SPM 2, a function implemented in NirsLab, to identify fNIRS channels that showed significant changes in HbO during the P300 task between the different groups.

#### 2.4.2. EEG

In our study, we used two approaches to analyze the EEG data, focusing on the P300 evoked potential. Using Letswave7 software, we first performed a permutation-based ANOVA to identify significant differences in ERP responses between subject groups. This method allowed us to assess statistical differences while maintaining strict control of the type I error rate by generating null distributions from permuted data.

We then implemented a non-parametric cluster-based permutation analysis [34], a robust technique for comparing ERP distributions between control groups and subjects with impaired aging in all conditions (static vs. dynamic * pleasant vs. unpleasant vs. neutral). This approach, which uses cluster statistics to maximize statistical power while maintaining adequate control over multiple comparisons, is based on the construction of clusters of adjacent electrodes that show statistically significant differences.

For the analysis, electrode neighbors were defined based on the physical distance to construct the neighbor matrix, and ERP averages for the static and dynamic conditions were calculated separately, aggregating data from all subjects for each condition.

A cluster-based analysis was performed on a time interval between 300 and 500 ms, focusing on differences between ERP averages. We identified clusters of electrodes that showed statistically significant differences using a critical value derived from the distribution of the maximum test statistics observed in the permutations.

## 3. Results

### 3.1. fNIRS Results

A Student’s *t*-test with a significance level of *p* < 0.05 was used for this analysis. The results revealed significant differences in the hemodynamic response to the P300 test, providing valuable insights into the neural dynamics underlying stimulus processing.

#### 3.1.1. Between Groups Comparison

**Figure 4:**
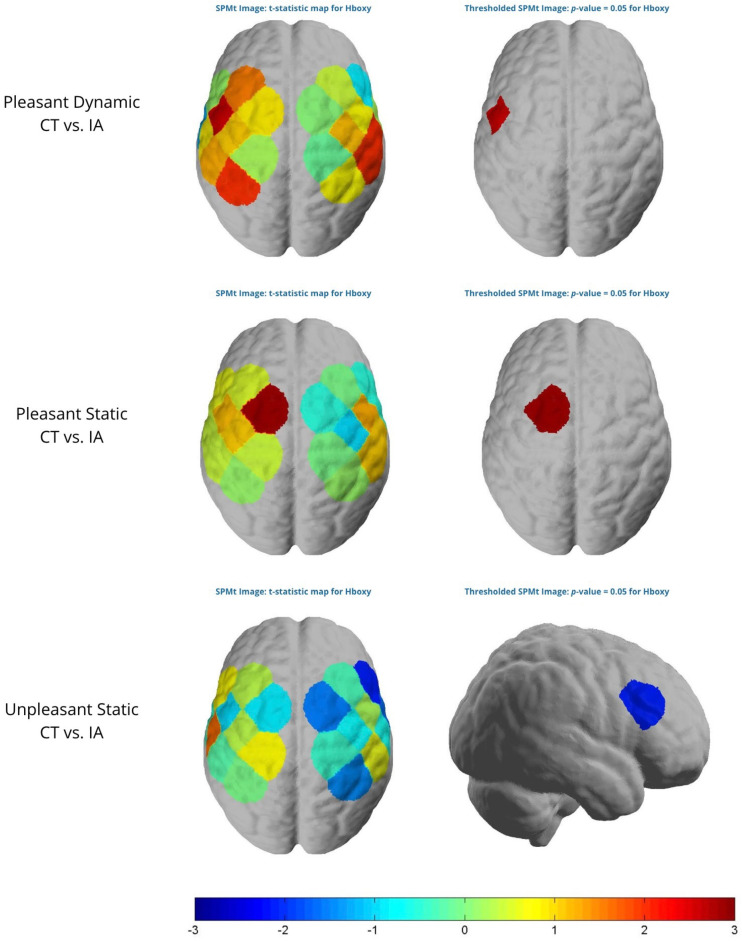
The analysis of oxyhemoglobin (HbO) is presented in two parts: the left side displays the raw beta value, while the right side shows the same values filtered to display only areas where brain activity is statistically significant (*p* < 0.05).

The analysis showed that patients had significantly higher HbO levels than controls in response to visual stimuli perceived as aesthetically pleasing, in both static (ch1, t = 2.90, *p* < 0.05) and dynamic (ch5, t = 2.61, *p* < 0.05) conditions, indicating greater brain activation (Figure 4). Conversely, controls showed higher HbO levels than patients in the static condition when presented with visual stimuli perceived as unpleasant. For neutral dynamic stimuli (ch10, t = 3, *p* < 0.05), the patients exhibited higher HbO levels than the controls, indicating differential responsiveness depending on the aesthetic value of the stimulus and condition.

#### 3.1.2. ANOVA

An ANOVA provided additional information on the interactions between group, condition, and aesthetic valence of the stimuli. Specifically, on channel 10, the interaction between group and aesthetic valence was significant (F(2) = 3.28, *p* = 0.042), indicating that the response to different types of aesthetic stimuli varies significantly between patients and controls (Table 1). Channel 20 showed a significant interaction between group (Table 2), condition, and aesthetic valence (F(2) = 3.45, *p* = 0.036). This suggests that the two groups’ brain activation is affected differently by the condition (static or dynamic) and their aesthetic valence.

### 3.2. EEG Results

**Figure 5:**
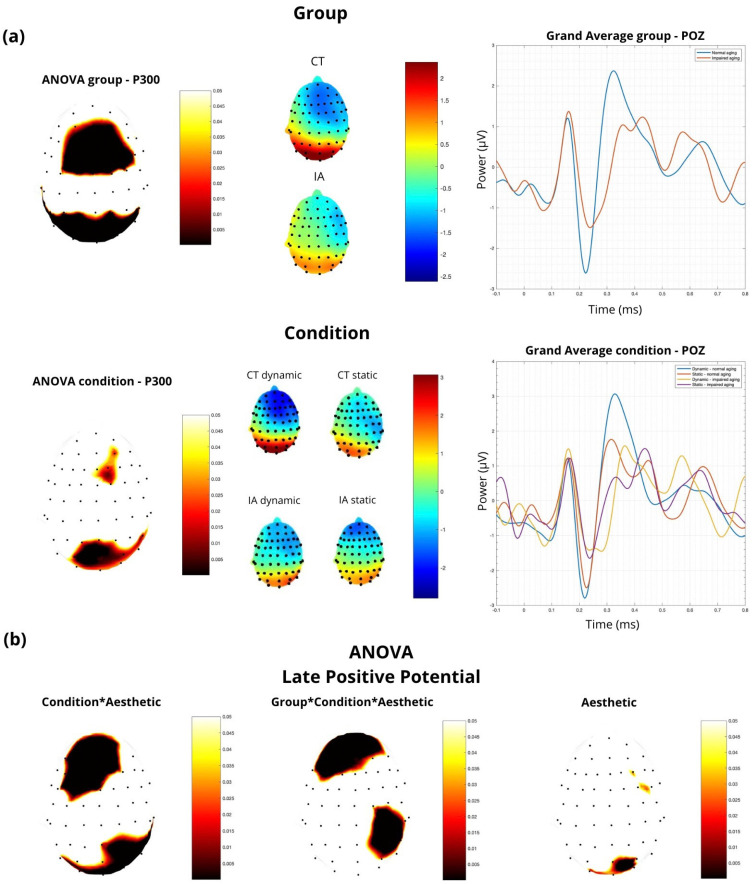
Statistical analysis and electrophysiological responses were used to evaluate aesthetic stimuli. (**a**) P300 ANOVA p-value in group and condition, showing the topographical map of raw activity and the wavelet; (**b**) LPP ANOVA p-value in condition*aesthetic (left), group*condition*aesthetic (central), and aesthetic (right).

In this study, we conducted an ANOVA to examine the effects of the interaction between condition, aesthetic appreciation, and group on ERP components, specifically on the late positive potential (LPP) and P300. The results indicated that the interaction between condition and aesthetic appreciation significantly influenced the LPP in the parietal region (POZ) (F = 3.29, *p* = 0.04; Figure 5). Additionally, the LPP also reached significance (F = 4.09, *p* = 0.019) in the prefrontal region (FPZ). Regarding image dynamism, significant modulation of P300 (F = 8.1, *p* = 0.005) and LPP (F = 5.24, *p* = 0.02) was observed in the POZ (Figure 5). This indicates that the experimental condition has a significant impact on brain activity measured by these metrics. The analysis also revealed that aesthetic appreciation has a significant effect on the LPP in the POZ (F = 3.67, *p* = 0.029; Figure 5), suggesting that aesthetic evaluations modulate brain activity in this area. The analysis revealed a significant association between the group factor and brain activity in the POZ (F = 14.1, *p* < 0.001; Figure 5), indicating substantial differences between groups. Additionally, a significant interaction effect was found between the group, experimental condition, and aesthetic appreciation in the FPZ and PO4 (F = 4.08, *p* = 0.019; Figure 5), suggesting that the relationship between brain activity and aesthetic appreciation is influenced by these factors.

#### Non-Parametric Cluster-Based Permutation Analysis

To evaluate the robustness of the results of the ANOVA, a non-parametric cluster-based permutation analysis was performed (Table 3).

**Figure 6:**
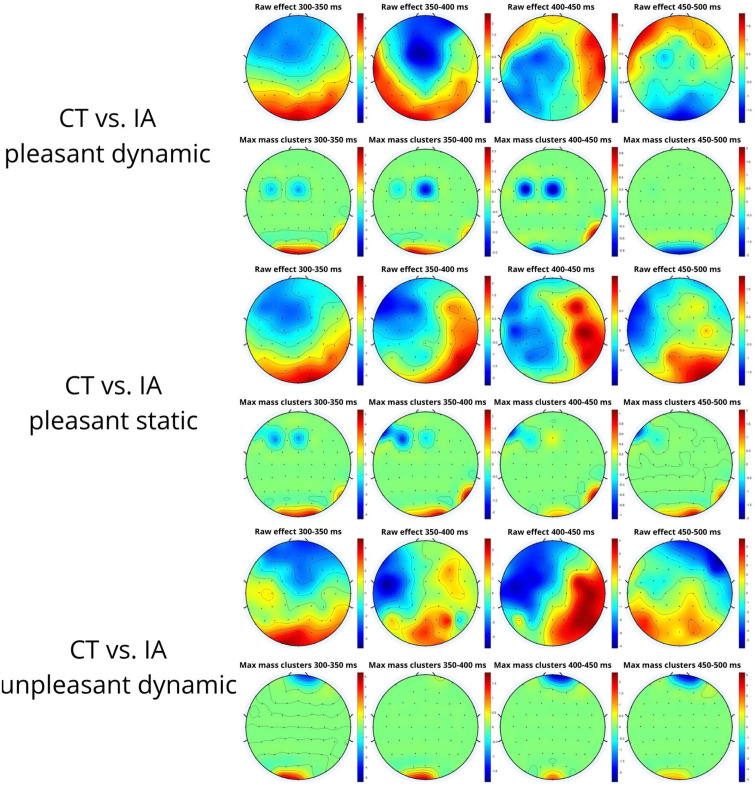
Non-parametric cluster-based permutation analysis showing topographical raw difference (top row) and raw difference filtered by cluster (down row) between groups in pleasant dynamic, pleasant static, and unpleasant dynamic stimuli.

When comparing the healthy control (CT) and impaired aging (IA) groups, significant clusters reflecting differences in P300 amplitude were identified. Positive clusters were observed for both pleasant (d = 2.10, Mass = 118.10) and unpleasant (d = 1.96, Mass = 18.21) dynamic stimuli, indicating that the CT group had a more pronounced P300 amplitude than the IA group (Figure 6). This suggests a more pronounced neurophysiological reactivity in controls, indicative of a greater ability to process dynamic stimuli, regardless of their aesthetic valence.

In the dynamic neutral stimuli, both positive (d = 2.05, Mass = 53.02) and negative (d = 2.05, Mass = 14.24) clusters were identified, revealing differences in P300 amplitude in the posterior area of the cortex (Figure 6). Between 300 and 350 ms, the positive cluster indicates that the CTs have a more pronounced P300 amplitude than the IA group. However, thereafter, the cluster becomes negative, suggesting a delay in latency response for the IA group.

## 4. Discussion

Our pilot study highlights the importance of multimodal neurophysiological assessment, particularly through the combined use of EEG and fNIRS, to investigate cognitive changes. The integration of these techniques provides a comprehensive view of neural dynamics, combining the strengths of each method to provide a more detailed and accurate understanding of brain function. This new BCI technology has gained popularity because it can more accurately decode brain activity in specific cortical regions while producing less electrical noise [35].

Recent literature highlights how the multimodal approach allows for a more holistic and detailed understanding of neural dynamics. This approach is particularly useful when studying complex neurological conditions [36,37], providing a richer and more multifaceted perspective that improves our ability to analyze and intervene in specific neurological changes.

To the best of our knowledge, this is the first study evaluating the EEG response to paintings in subjects with impaired aging.

### 4.1. fNIRS

Our investigation using functional near-infrared spectroscopy (fNIRS) revealed significant differences in brain activation between individuals with impaired aging and healthy controls. The study highlights how the nature of visual stimuli, both static and dynamic, affects brain activation in distinct ways.

Specifically, we observed that the group with impaired aging exhibited significantly higher levels of oxyhemoglobin (HbO) than controls in response to aesthetically pleasing stimuli, indicating greater brain activation. This increase in activation in patients can be explained by the neural inefficiency theory [38], which suggests that individuals with cognitive impairment may require more brain activation to perform the same cognitive tasks as healthy controls. According to this theory, individuals with neurological conditions that result in impaired cortical functioning, such as multiple sclerosis, Parkinson’s disease, and mild cognitive impairment, must compensate for their neural inefficiencies by increasing activation in specific brain areas to maintain a comparable level of behavioral performance [39,40,41]. The heightened activation observed in response to aesthetically pleasing stimuli may indicate the brain’s effort to compensate for challenges in processing such stimuli, which necessitate more intricate interpretation and appreciation.

The evaluation of beauty, indeed, involves complex cognitive processing that engages multiple brain areas [42]. This process is known as the ‘aesthetic triad’ and comprises “neural systems that contribute to emergent aesthetic experience. Aesthetic experiences are emergent states resulting from interactions between sensory-motor, emotional-evaluative, and meaning-knowledge neural systems” [42]. Furthermore, our findings align with previous studies that have explored the processing of complex stimuli in individuals with mild cognitive impairment (MCI). For instance, Niu et al. [43] and Yeung et al. [44] have demonstrated that patients with MCI display modified brain activation patterns when performing tasks that require a greater working memory load.

### 4.2. EEG

In our EEG results, we observed significant differences in P300 amplitude between groups of subjects with impaired aging and healthy controls, particularly in response to aesthetic stimuli.

Neurophysiological markers, such as EEG and event-related potential (ERP) components, have been identified as significant indicators of cognitive decline in the scientific literature [45]. They offer valuable tools for determining pathological stages [46]. P300, in particular, emerges as a key ERP component for the study of attention, working memory, and cognitive impairment. P300 is a positive peak that is prominent in the centro-parietal areas of the scalp. It usually occurs between 250 and 500 ms after stimulus presentation. The first subcomponent of P300, P3a, is related to attention mechanisms and the handling of novel stimuli [47]. Research has shown that changes in P300 latency and amplitude may indicate the progression of neurodegeneration [48], showing a longer latency and a more attenuated amplitude [49]. Other studies have confirmed significant differences in P300 parameters between healthy individuals and those with MCI or Alzheimer’s Disease (AD) [50,51].

Based on the evidence discussed above, these differences appear to be consistent with studies that have reported reduced P300 amplitude in patients with MCI compared to healthy controls in both auditory [52] and visual [53] tasks, suggesting an alteration in cognitive processing ability in this population. The study’s findings suggest that the processing of aesthetically pleasing stimuli requires a complex set of cognitive functions, which may be impaired in individuals with impaired aging.

Indeed, aesthetic appreciation is a complex process that involves perception, integration of implicit memory, explicit classification, cognitive mastery, and evaluation [54]. This process is also influenced by additional factors, such as the environment and social interaction. According to our data, healthy controls show greater amplitude in response to stimuli perceived as pleasant compared to the impaired aging group. This suggests a greater sensitivity or ability to process aesthetic stimuli and supports the integrity of the previously discussed processes. In contrast, the group of patients exhibited greater activation in response to neutral stimuli than to aesthetically pleasing stimuli. This could indicate a reduced ability to discriminate aesthetics and process the aesthetic qualities of stimuli, and/or deficits in one or more of those processes [55].

In our study, the LPP, which is associated with emotional and evaluative processing of stimuli, exhibited a more right-lateralized distribution when comparing the CT and IA groups, consistent with previous studies that have shown that aesthetic judgments can elicit a stronger distribution in the right hemisphere of the brain [56]. This statement is in line with the literature that links the LPP to a heightened response to objects with high affordance and aesthetic levels [57]. This suggests that objects that are both highly attractive and perceived as functional elicit a privileged neural response.

### 4.3. Limitations

Although this pilot study provides significant insights into the combined use of EEG and fNIRS to explore neurophysiological differences associated with aesthetic appreciation in individuals with impaired aging, it has some limitations. The small sample size limits the generalizability of our results, suggesting the need for future studies with larger samples to confirm and expand our findings. The assessment of aesthetic appreciation, which is inherently subjective, may have been influenced by uncontrolled factors, such as cultural and personal preferences. Additionally, the interpretation of ERP data, particularly the P300 data, despite the extensive literature present, requires caution, as alterations may be attributable to a variety of factors. Finally, this study highlights the potential of BCIs in the clinical setting, but further research is needed to refine interfaces and analysis techniques for targeted clinical applications.

## 5. Conclusions

This pilot study highlights the relevance of a multimodal approach combining EEG and fNIRS to investigate the neural dynamics associated with aesthetic appreciation and impaired aging, demonstrating significant differences in brain activation between the two groups in response to aesthetic stimuli. Although the group with impaired aging showed difficulty in the P300-related task compared to the control group, the results suggest compensation in terms of hemodynamic recruitment. This finding could provide a basis for cognitive rehabilitation by adjusting the environment according to the subjects’ aesthetic perception to improve outcomes.

The combined use of non-invasive BCI technologies is a valuable tool for neurophysiological assessment, contributing to the emerging literature on their use in clinical settings. The results highlight the potential of these technologies in improving the diagnosis and understanding of cognitive disorders. The ability to detect these dynamics only through the multimodal use of BCIs emphasizes the effectiveness of integrating different methodologies for a more comprehensive understanding of the neural processes involved in aesthetic appreciation and cognitive impairment.

## Figures and Tables

**Table 1:** fNIRS Channel 10: ANOVA on group, condition, and aesthetic appreciation.

*ch 10*	*Sum of Squares*	*df*	*Mean Square*	*F*	*p*
*group*	4.37 × 10^−7^	1	4.37 × 10^−7^	1.63	0.205
*condition*	4.76 × 10^−7^	1	4.76 × 10^−7^	1.77	0.186
*aesthetic*	1.02 × 10^−6^	2	5.08 × 10^−7^	1.89	0.156
*group x condition*	6.08 × 10^−7^	1	6.08 × 10^−7^	2.27	0.135
*group x aesthetic*	1.76 × 10^−6^	2	8.80 × 10^−7^	3.28	0.042 *
*condition x aesthetic*	1.07 × 10^−6^	2	5.36 × 10^−7^	2.00	0.141
*group x condition x aesthetic*	1.20 × 10^−6^	2	5.98 × 10^−7^	2.23	0.113
*Residuals*	2.63 × 10^−5^	98	2.68 × 10^−7^		

Note: * *p* < 0.05.

**Table 2:** fNIRS Channel 20: ANOVA on Group, Condition, and Aesthetic appreciation.

*ch 20*	*Sum of Squares*	*df*	*Mean Square*	*F*	*p*
*group*	2.30 × 10^−8^	1	2.30 × 10^−8^	0.06	0.809
*condition*	9.68 × 10^−7^	1	9.68 × 10^−7^	2.47	0.119
*aesthetic*	3.99 × 10^−8^	2	2.00 × 10^−8^	0.05	0.950
*group x condition*	1.48 × 10^−6^	1	1.48 × 10^−6^	3.78	0.055
*group x aesthetic*	9.25 × 10^−8^	2	4.62 × 10^−8^	0.12	0.889
*condition x aesthetic*	2.22 × 10^−6^	2	1.11 × 10^−6^	2.84	0.063
*group x condition x aesthetic*	2.70 × 10^−6^	2	1.35 × 10^−6^	3.45	0.036 *
*Residuals*	3.83 × 10^−5^	98	3.91 × 10^−7^		

Note: * *p* < 0.05.

**Table 3:** Summary of cluster-based permutation analysis: Cohen’s d, cluster Mass, and raw effect differences of clusters.

		*Max Positive Cluster*			*Max Negative Cluster*		
	Cohen’s D	Mass	ERP (Mean)	ERP (sd)	Mass	ERP (Mean)	ERP (sd)
*Pleasant dynamic CT* vs. *IA*	2.10	118.10	3.37	3.24	102.92	0.20	1.55
*Pleasant static CT* vs. *IA*	2.27	128.42	2.80	4.83	88.80	−0.69	2.76
*Unpleasant dynamic CT* vs. *IA*	1.96	38.21	0.57	5.06	16.06	1.30	8.12
*Unpleasant static CT* vs. *IA*	2.33	34.67	−1.31	1.57	73.25	1.52	4.18
*Neutral dynamic CT* vs. *IA*	2.05	53.02	4.44	4.45	14.24	1.37	6.17
*Neutral static CT* vs. *IA*	1.76	21.31	−0.65	2.26	27.98	0.95	3.15
*Control dynamic pleasant* vs. *unpleasant*	2.18	11.40	−0.48	2.09	34.99	0.06	2.36
*Control dynamic pleasant* vs. *neutral*	-	-	-	-	-	-	-
*Control dynamic unpleasant* vs. *neutral*	1.95	16.85	0.06	2.36	7.13	−3.48	1.07
*Patient dynamic pleasant* vs. *unpleasant*	2.12	130.55	1.37	1.31	75.97	0.28	6.43
*Patient dynamic pleasant* vs. *neutral*	1.81	41.33	0.46	1.64	19.79	4.17	7.08
*Patient dynamic unpleasant* vs. *neutral*	2.04	11.98	−0.28	4.73	30.83	1.98	6.07
*Control static pleasant* vs. *unpleasant*	2.15	20.29	0.21	1.94	7.66	−0.76	2.41
*Control static pleasant* vs. *neutral*	2.10	15.41	2.25	3.14	9.44	0.56	1.03
*Control static unpleasant* vs. *neutral*	2.82	20.15	0.69	3.99	17.51	−1.70	0.94
*Patient static pleasant* vs. *unpleasant*	2.32	24.60	−1.47	2.62	46.32	0.40	3.31
*Patient static pleasant* vs. *neutral*	1.75	26.75	−1.17	2.65	12.06	0.42	3.66
*Patient static unpleasant* vs. *neutral*	2.16	27.86	0.10	5.33	22.55	0.42	3.66
*Control pleasant dynamic* vs. *static*	1.62	15.38	−3.57	2.98	14.80	−0.85	2.60
*Control unpleasant dynamic* vs. *static*	2.45	61.84	1.62	4.66	21.37	−1.60	2.18
*Control neutral dynamic* vs. *static*	2.23	16.94	0.37	1.60	24.52	−1.85	1.75
*Patient pleasant dynamic* vs. *static*	1.57	31.34	0.71	1.04	14.01	−1.86	2.42
*Patient unpleasant dynamic* vs. *static*	2.51	60.60	0.22	5.44	57.08	0.93	4.19
*Patient neutral dynamic* vs. *static*	1.49	27.86	4.36	5.26	33.90	0.32	3.33

## Data Availability

The raw data supporting the conclusions of this article will be made available by the authors, without undue reservation.
